# Impact of primary healthcare providers’ initial role security and therapeutic commitment on implementing brief interventions in managing risky alcohol consumption: a cluster randomised factorial trial

**DOI:** 10.1186/s13012-016-0468-5

**Published:** 2016-07-16

**Authors:** M. Keurhorst, P. Anderson, M. Heinen, Preben Bendtsen, Begoña Baena, Krzysztof Brzózka, Joan Colom, Paolo Deluca, Colin Drummond, Eileen Kaner, Karolina Kłoda, Artur Mierzecki, Dorothy Newbury-Birch, Katarzyna Okulicz-Kozaryn, Jorge Palacio-Vieira, Kathryn Parkinson, Jillian Reynolds, Gaby Ronda, Lidia Segura, Luiza Słodownik, Fredrik Spak, Ben van Steenkiste, Paul Wallace, Amy Wolstenholme, Marcin Wojnar, Antoni Gual, M. Laurant, M. Wensing

**Affiliations:** 1 Radboud Institute for Health Sciences, Radboud Center for Quality of Healthcare (IQ healthcare), Radboud university medical center, Nijmegen, The Netherlands; 2 Centre for Nursing Research, Saxion University of Applied Sciences, Deventer, Enschede The Netherlands; 3 Institute of Health and Society, Newcastle University, Newcastle, England, UK; 4 Department of Family Medicine, School CAPHRI, Maastricht University, Maastricht, The Netherlands; 5 Department of Medical Specialist and Department of Medicine and Health Sciences, Linköping University, Motala, Sweden; 6 Program on Substance Abuse, Public Health Agency, Government of Catalonia, Barcelona, Spain; 7 State Agency for Prevention of Alcohol-Related Problems, Warsaw, Poland; 8 National Addiction Centre, Institute of Psychiatry, Psychology and Neuroscience, King’s College London, London, UK; 9 Independent Laboratory of Family Physician Education, Pomeranian Medical University in Szczecin, Szczecin, Poland; 10 School of Health & Social Care, University of Teesside, Middlesbrough, UK; 11 Hospital Clínic de Barcelona, Fundació Clínic per a la Recerca Biomèdica, Barcelona, Spain; 12 Department of Social Medicine, University of Gothenburg, Gothenburg, Sweden; 13 Department of Primary Care and Population Health, University College London, London, UK; 14 Medical University of Warsaw, Warsaw, Poland; 15 Hospital Clínic de Barcelona, IDIBAPS, Barcelona, Spain; 16 Faculty of Health and Social Studies, HAN University of Applied Sciences, Nijmegen, The Netherlands; 17 Department of General Practice and Health Services Research, University Heidelberg Hospital, Heidelberg, Germany

**Keywords:** Brief interventions, Risky drinking, Primary healthcare, Provider influences, Implementation research

## Abstract

**Background:**

Brief interventions in primary healthcare are cost-effective in reducing drinking problems but poorly implemented in routine practice. Although evidence about implementing brief interventions is growing, knowledge is limited with regard to impact of initial role security and therapeutic commitment on brief intervention implementation.

**Methods:**

In a cluster randomised factorial trial, 120 primary healthcare units (PHCUs) were randomised to eight groups: care as usual, training and support, financial reimbursement, and the opportunity to refer patients to an internet-based brief intervention (e-BI); paired combinations of these three strategies, and all three strategies combined. To explore the impact of initial role security and therapeutic commitment on implementing brief interventions, we performed multilevel linear regression analyses adapted to the factorial design.

**Results:**

Data from 746 providers from 120 PHCUs were included in the analyses. Baseline role security and therapeutic commitment were found not to influence implementation of brief interventions. Furthermore, there were no significant interactions between these characteristics and allocated implementation groups.

**Conclusions:**

The extent to which providers changed their brief intervention delivery following experience of different implementation strategies was not determined by their initial attitudes towards alcohol problems. In future research, more attention is needed to unravel the causal relation between practitioners’ attitudes, their actual behaviour and care improvement strategies to enhance implementation science.

**Trial registration:**

ClinicalTrials.gov: NCT01501552

**Electronic supplementary material:**

The online version of this article (doi:10.1186/s13012-016-0468-5) contains supplementary material, which is available to authorized users.

## Background

The World Health Organization Status Report on Alcohol and Health documented that the level of alcohol consumption in the European Union (EU) is almost double the global average, as on average almost 2.5 alcoholic drinks (25 g) per capita are consumed every day [1]. Alcohol is an attributable cause of more than 200 three-digit International Classification of Disease (ICD)-10 codes [2] and, in the age group of 15–49 years, it is the leading risk factor for the global burden of disease [3]. In Europe, one in every seven deaths in men and one in every 13 deaths in women in the group aged 15–64 years is due to alcohol consumption [3].

In a Cochrane review, Kaner et al. showed that screening and brief interventions (SBI) in primary healthcare to detect and intervene in risky alcohol consumption are cost-effective in reducing alcohol consumption with a mean difference of −38 g/week after a year of follow-up. Included studies were performed in English-speaking countries (UK, USA, Canada, Australia) and various European countries. No studies were based in transitional or developing countries [4]. However, SBI are poorly implemented in primary healthcare settings [5–8]. The reasons for this include providers’ lack of knowledge, low role security and therapeutic commitment, lack of financial resources and lack of time [9, 10]. Therapeutic commitment refers to motivation, task-specific self-esteem and work satisfaction towards patients with risky alcohol consumption. Role security refers to skills, knowledge and owning a role of working with risky drinkers [11]. Previous research has shown that general practitioners (GPs) with greater role security and therapeutic commitment towards patients with risky alcohol consumption report being more involved in managing alcohol-related problems than others [12, 13]. In due course, there have been several studies undertaken on methods to overcome these barriers with implementation strategies to embed SBI in routine care [14–16]. Van Beurden et al. (2012), for instance, described that one of the reasons for failing in improving SBI was sub-optimal implementation of the programme [14]. The study of Funk et al. concluded that adopting more direct dissemination approaches for SBI evidence-based programmes to GPs is a necessary first step for changing practice behaviour [15]. These examples of former studies, of which some we conducted on a large scale, showed what worked sub-optimally; however, they did not show yet what actually did work. Consequently, evidence for optimally designed implementation strategies for a wider uptake of SBI remains inconclusive [17]. To date, it is unclear which provider characteristics influence implementation of brief interventions for risky alcohol consumption. More specifically, it remains unclear to what extent primary healthcare providers’ role security and therapeutic commitment impact on the brief intervention implementation. A recent survey in eight European countries showed that physicians who had received more education on alcohol and who had higher role security and therapeutic commitment reported managing a higher number of patients for alcohol and alcohol problems [10]. If providers’ baseline role security and therapeutic commitment are not accounted for in implementation programmes, interventions can lead to a deterioration in role security and therapeutic commitment [12]. However, another trial showed a lack of improvement in role security and brief intervention delivery despite the extended implementation programme that included addressing providers’ role security and therapeutic commitment [18]. So far, it still is unclear whether positive attitudes are required to improve role security and therapeutic commitment for improving screening and brief intervention delivery.

With more knowledge on the impact of role security and therapeutic commitment, one may be able to adapt to and take these into account within implementation programmes. The Optimizing Delivery of Healthcare Interventions (ODHIN; www.odhinproject.eu) trial increases our knowledge on the best methods for improving brief interventions’ frequency in primary healthcare. This trial concerned a multifaceted programme to implement brief interventions in routine primary healthcare, using specific training and support on how to deal with alcohol-related problems, financial reimbursement and the opportunity to refer screen-positive patients to an internet-based brief intervention (e-BI) as strategies. The trial showed that the highest increase in screening and brief intervention activity was present in the primary healthcare units (PHCUs) that received training and support combined with financial reimbursement [19].

Based on the current evidence base, we hypothesised that higher baseline role security and therapeutic commitment might positively influence the impact of training and support and financial reimbursement on screening and brief advice [10, 18]. Therefore, the aim of this paper was to evaluate whether providers’ initial role security and therapeutic commitment impacted the eight strategy types on implementing brief alcohol interventions in PHCU across five countries in the European Union.

## Methods

This paper builds on the findings from the ODHIN trial, which was focused on identifying implementation strategies for implementing brief interventions [19]. Therefore, the methods section presents the main key ingredients of the ODHIN study. More information can be found in another ODHIN paper that presents the trial effects on screening and brief intervention activity. CONSORT guidelines were followed in reporting the trial [20].

### Study design and participants

ODHIN was a cluster randomised 2 × 2 × 2 factorial trial as described in the study protocol (ClinicalTrials.gov. Trial identifier: NCT01501552) [21]. English, Catalan, Polish, Swedish and Dutch PHCUs participated and combined their data to examine the effect of three different implementation strategies (training and support, financial reimbursement and referral opportunities to an internet-based brief intervention programme) on screening and brief intervention activity for risky drinkers identified by screening using the AUDIT-C questionnaire screening tool [22].

### Implementation strategies

After formal agreement of the PHCUs to take part in the trial, a four-week baseline measurement took place, during which no trial interventions were administered. After a 2- to 6-week gap, the 12-week implementation period occurred with the start date for each country between November 2012 and May 2013. All eight allocation groups received the same input as controls but with additional components added.

1. Control group: care as usual
2. Training and support (TS)
3. Financial reimbursement (FR)
4. Referral to internet-based brief interventions (e-BI)
5. TS and FR
6. TS and e-BI
7. FR and e-BI
8. TS, FR and e-BI

More details about the implementation strategies and procedural activities were described in Additional file 1 [19, 21].

### Measures

#### Brief intervention proportions

Brief intervention proportions were the primary outcome of the ODHIN study. These were measured during two time frames: during the four-week baseline period and during the 12-week implementation period. The providers completed paper tally sheets (Catalonia applied electronic tally sheets). The tally sheets included AUDIT-C questions, AUDIT-C scores and tick boxes to indicate the type of intervention (oral advice, an advice leaflet, referral to the e-BI programme, or referral for advice to another provider in or outside the PHCU) that was delivered.

The brief intervention proportions were calculated as a number of AUDIT-C positive patients that received one or more of oral advice, an advice leaflet, referral to the e-BI programme, or referral for advice to another provider in or outside the PHCUs, divided by the total number of adult (≥18 years) consultations of the participating providers per PHCU. We added up all the patient consultations in the 12-week implementation period and calculated accordingly the proportion of AUDIT-C positive patients that received a brief intervention. Impact of the implementation strategies on screening and brief advice activity was reported elsewhere [19].

#### Role security and therapeutic commitment

Before starting the baseline measurement of brief interventions, providers completed a questionnaire in which they provided their demographical features, including gender, age and occupation (e.g. medical practitioner, nurse, practice assistant).

Role security and therapeutic commitment were measured at baseline by the short version of the Alcohol and Alcohol Problems Perception Questionnaire (SAAPPQ), translated into the native language of each participating country [11]. The SAAPPQ is a validated instrument based on factor analysis [11] of the original tool as developed and validated by Cartwright [23]. The shortened version of the original attitude scale has been demonstrated to be as representative and discriminatory as the full AAPPQ scale [11]. The SAAPPQ has good test-retest reliability and Cronbach’s alpha in the range of 0.7 and 0.9 [11]. Furthermore, it has been applied in several international contexts and with various disciplines (e.g. [24–27]). All participating providers who provided written informed consent were asked to complete the SAAPPQ. Role security measures role adequacy, for example “I feel I can appropriately advise my patients about drinking and its effects”, and role legitimacy, for example, “I feel I have the right to ask patients questions about their drinking when necessary”. Role security is expressed at the emotional level, whereas therapeutic commitment measures motivation, for example “pessimism is the most realistic attitude to take toward drinkers”; task specific self-esteem, for example “all in all I am inclined to feel I am a failure with drinkers”; and work satisfaction, for example “in general, it is rewarding to work with drinkers”. Role security includes four items on a seven-point Likert scale and summed scores range between 4 and 28. Therapeutic commitment includes six items on a seven-point Likert scale and summed scores range between 6 and 42.

### Sample size and randomisation

To achieve sufficient statistical power for significant effects on intervention proportions, it was estimated that 120 PHCUs (15 per eight allocation groups, evenly distributed between countries) would be needed [21].

Randomisation took place after formal agreement of the PHCUs to take part in the trial. The PHCUs were randomly allocated to one of the eight allocated groups by the ODHIN coordinating centre, using computerised randomisation, stratified by country (i.e. block randomisation), ensuring 15 PHCUs per group (three per group in each country) [21].

### Statistical analysis

Because of the hierarchical structure (providers nested within PHCU, nested within country), we performed a two-level linear multilevel analysis (mixed model). We performed a model with random intercept for countries and practices, and other variables such as TS, FR, e-BI, and baseline brief intervention proportion fixed. The outcome measure was brief intervention proportion after the implementation period. Multiple imputation was not applied as the percentage of missing cases was 1.5 % [28].

When examining the impact of baseline role security and therapeutic commitment on the 12-week brief intervention proportions, examination of residuals found them to be not symmetrically distributed around zero, so the data underwent log transformation, which provided a better fit. Prior to log transformation, proportions with a value of zero were assigned a value of 0.001. Coefficients for the combined effects of TS + FR and TS + e-BI were the sum of the individual coefficients. Since the data were log transformed, the contrast coefficients are relative effects. The percentage difference in brief intervention proportions with each implementation strategy as opposed to without, was calculated with the equation: difference (%) = 100 × (exp)2 × coefficient estimate from procedure MIXED minus 1. As this approach creates some issues with outcomes with a zero value, 0.001 was added to each proportion. In order to test the validity of this assumption, a sensitivity analysis was undertaken using the exact proportions but excluding those PHCU with an outcome of zero.

Correlations between the eight variables were tested with Pearson’s correlation test. To test the influence of baseline role security and therapeutic commitment on the delivered brief interventions, the model was run with both provider characteristics, included one by one. When both characteristics significantly correlated, which was expected as they were included in the same questionnaire, they were paired when entered included in the statistical model. Furthermore, for both role security and therapeutic commitment, we added interaction terms in order to identify interactive effects of characteristics with the implementation strategies. We considered a *p* value <0.05 statistically significant. In case of interaction, subgroups of variables were analysed separately. The statistical analyses were performed with IBM SPSS v20. The datasets are available upon request.

## Results

### Study population

**Fig. 1:**
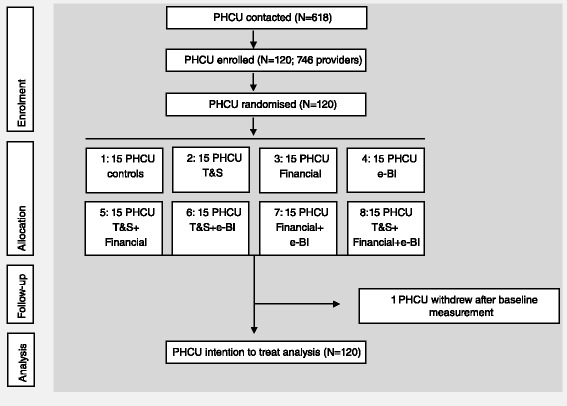
Trial flow chart

Figure 1 outlines the flow of participating PHCUs and providers throughout the parent trial. The 120 participating PHCUs with 746 providers were randomised and included in the analyses. Table 1 shows baseline characteristics of the participating providers. Almost three quarters of the participating providers were women and the mean age of all participating providers was 47.0 years (SD 9.4). Occupations of participants varied, though participants were mainly GPs or nurses. Most participating providers were employed in health clinics and group practices. The mean number of consulting adult patients per month per provider during the baseline was 242, but varied greatly between providers with a standard deviation of 188. Role security was high at baseline, with a score of 21.0 (SD 3.5) within a possible range of 4–28. Therapeutic commitment scored on average 27.2 (SD 4.7) within a possible range of 6–42. There were no baseline differences observed between any of the eight allocation groups; however, PHCU type, the number of registered patients in the PHCU, and the number of consulting adult patients per provider were significantly correlated with each other (health centres had the highest number of registered patients and consultations, and solo practices the lowest) (all *p* < 0.05).

**Table 1:** Characteristics of participating providers

Characteristic	All participants (*n* = 746)^d^
*N* (%) of women	559 (74.9)
Mean (SD) age in years	47.0 (9.4)
Occupation (%)	
-GP	54.7
-Nurse	37.8
-Practice assistant	5.1
-Other	2.3
Type of PHCU^a^ (%)	
-Solo	26 PHCUs (21.7)
	72 providers (9.7)
-Duo	13 PHCUs (10.8)
	67 providers (9.0)
-Group	23 PHCUs (19.2)
	-132 providers (17.7)
-Health clinic	-58 PHCUs (48.3)
	-475 providers (63.7)
Baseline mean role security^b^ (SD)	21.0 (3.5)
Baseline mean therapeutic commitment^c^ (SD)	27.2 (4.7)

^a^ *PHCU* primary healthcare unit. In Poland, providers normally operate as single-handed entities working with other providers in one building, three providers and their staff working in one building was regarded as one PHCU

^b^Score at minimum 4; at maximum 28

^c^Score at minimum 6; at maximum 42

^d^No differences in baseline measures. The analyses to check for differences in baseline measures between allocation groups took into account the nested nature of the data

### Influence of baseline role security and therapeutic commitment on implementation

Baseline role security and therapeutic commitment were significantly correlated with a Pearson correlation of 0.379 (*p* < 0.001). This was accounted for in the analytical model by adding these as a pair to the statistical model. Testing the influence of providers’ role security and therapeutic commitment towards dealing with risky drinking prior to the trial, that is, their baseline role security and therapeutic commitment, taking account for the correlation between these characteristics, showed no change in coefficients (i.e. not more than 10 % change) of impact of the implementation strategies on brief intervention implementation (Table 2). Furthermore, there were no significant interactions between these characteristics and allocated groups nor a difference in impact between the provider groups with low (below median score) or high levels (above median score).

**Table 2:** Impact of baseline role security and therapeutic commitment on the relative per cent difference in the proportion of patients receiving an intervention during the 12-week study period between providers that received the implementation component and providers that did not receive the implementation component

Implementation component	Basic model: brief intervention proportion difference (95 % CI; *p* value)^a^		Basic + RS + TC: brief intervention proportion difference (95 % CI; *p* value)^a^	
Training and support (TS)	60.4	(14.5–124.8; 0.007)	62.5	(15.9–127.9; 0.005)
Financial reimbursement (FR)	68.8	(20.5–136.6; 0.003)	67.4	(19.4–134.7; 0.003)
e-BI	4.9	(−25.1–47; 0.78)	5.8	(−24.5–48.3; 0.741)
TS plus FR	170.7	(68.7–334.6; <0.001)	172.0	(69.3–337; <0.001)
TS plus e-BI	68.3	(4.1–172.2; 0.034)	71.9	(6.2–178.4; 0.028)
FR plus e-BI	77.1	(10.6–183.7; 0.018)	77.1	(10.6–183.7; 0.018)
TS plus FR plus e-BI	184.1	(59.2–407.0; 0.001)	187.8	(61.2–414; <0.001)

*RS* role security; *TC* therapeutic commitment

^a^Adjusted for baseline brief intervention rates and accounting for providers nested within PHCU nested within country

Sensitivity analysis suggested the addition of 0.001 to the observed outcome of a zero value. In order to test the validity of this assumption, a sensitivity analysis was undertaken using the exact proportions but excluding those PHCU with an outcome of zero.

## Discussion

The aim of this paper was to evaluate whether providers’ initial role security and therapeutic commitment impacted upon the eight intervention strategies, which aimed to promote implementation of brief alcohol interventions in PHCU across five countries in the European Union. Baseline role security and therapeutic commitment both appeared to have no influence on implementation of brief interventions in this study, by neither training and support (TS), financial reimbursement (FR), nor the opportunity to refer patients to an internet-based brief intervention (e-BI). This may suggest that applied implementation strategies overwhelmed attitudes to be conditional for improved screening and brief intervention delivery.

The results of this study indicate that initial role security and therapeutic commitment will not play a significant role in the implementation process, including the FR and TS strategies that were effective in raising brief interventions. Factors that actually do play a significant role in improvement of brief interventions by FR are still unknown. In this study, the financial reimbursement scheme differed per country. In Poland and Catalonia, providers were reimbursed directly, whereas in Sweden and the Netherlands, reimbursement was applied at a PHCU level [21]. Nevertheless, in both reimbursement schemes, FR could be regarded as an external brief intervention motivator. We cannot explain the process behind FR successes on country level, as it could not be the country itself causing the effect, because the multilevel analysis model accounted for providers nested within PHCUs, nested within the country. Reviews support the view of these still unexplained processes behind FR implementation successes [29]. Therefore, more research about implementation processes in this context is needed, preferably to conduct a meta-analysis to enhance quality of evidence, since besides the ODHIN study there are very few studies that tested FR effects on alcohol brief intervention delivery. Additionally, it is also reported that a pay for performance approach can be used to improve the quality of care; however, it is not a “magic bullet” as it works optimally as part of a wider implementation strategy programme [30].

The hypothesis that baseline provider’s role security and therapeutic commitment have a positive impact on the number of patients managed for their risky alcohol consumption [10, 12, 13] was not confirmed by this study. Furthermore, in this study, the training and support strategy is both effective for providers with low and high baseline role security and therapeutic commitment, in contrast to the findings of Anderson et al. (2004) where attitudinal levels deteriorated even for those with low levels at baseline [12]. Moreover, compared to the Anderson et al. study [12], role security and therapeutic commitment were relatively high in the ODHIN study. Having this combined with standard deviations of 3.5 and 4.7 on means of, respectively, 21 and 27.2 may reduce the likelihood of identifying these variables as moderating characteristics. In addition, as practitioner brief intervention delivery was significantly improved in this study, the results question the importance of role security and therapeutic commitment in the implementation process. However, besides possible ceiling effects of the instrument measuring these attitudinal constructs, we must acknowledge that in this study only baseline role security and therapeutic commitment was included, while these can evolve over time. So, our finding does not rule out any importance of these characteristics but merely indicate that the extent to which providers managed to change their brief intervention proportions when submitted to different implementation strategies was not determined by their initial attitudes towards alcohol problems. In future research, more attention is needed whether importance of role security and therapeutic commitment can be ruled out when implementing alcohol brief interventions, as it can inform us whether to focus on these or not in implementation trajectories.

The study had strengths and limitations. The implementation strategies were applied in all five countries, but the specific content was tailored to the country context. To preserve comparability between countries, we formulated minimum requirements that country-specific implementation strategies had to meet. For the remainder, countries had flexibility in making the strategies compatible with country standards. Therefore, we think that these results are valuable for policy makers from different European countries, especially with outcomes of this process analyses. Another issue that deserves discussion is the enrolment rate of 19 %. Only one in five practices consented to participate, which may indicate that only motivated providers participated. This suggests that implementation strategy effects may be lower as reported in the ODHIN study. A strength was the hierarchical structure of individual providers being nested within PHCU, and PHCU being nested within country, which was taken into account in the analyses. Lastly, the five participating countries differ in their organisation of primary healthcare and in their burden of alcohol consumption as well as their drinking patterns. Therefore, our findings could be generalised to other western countries as well.

A limitation of the study is the tally sheet to measure AUDIT-C outcomes, that may be a source of bias. Providers could check boxes for interventions being done, without the researchers’ check if the intervention actually was carried out. Besides, the tally sheet already included options of interventions, which may have been an intervention in itself. Another weakness of the study is the lack of results concerning the country-specific effects of our implementation strategies, as the study was powered on the total of five countries. Consequently, the cross-cultural differences between the countries were not addressed. However, this was not the primary goal of this international study. An ideal study design would be a design with sufficient power to assess inter-country differences. Furthermore, in order to make the design less difficult to interpret, instead of a factorial design, a stepped wedge could be selected to assess comparable implementation strategies as evaluated in the ODHIN study.

## Conclusions

In this study, providers’ baseline role security and therapeutic commitment had no discernible impact on implementing brief interventions. The extent to which providers managed to change their brief intervention proportions when subjected to different implementation strategies was not determined by their initial attitudes towards alcohol problems. In future research, more attention is needed to unravel the causal relation between practitioners’ attitudes, their actual behaviour and care improvement strategies to enhance implementation science, as it can inform us whether to focus on these or not in implementation trajectories.

## Abbreviations

AUDIT-C, alcohol use disorders identification test, comprehensive version; e-BI, referral to internet-based brief interventions; EU, European Union; FR, financial reimbursement; GP, general practitioner; ICD, International Classification of Disease; ODHIN, Optimizing Delivery of Healthcare Interventions; PHCU, primary healthcare unit; SAAPPQ, short version of the Alcohol and Alcohol Problems Perception questionnaire; SBI, screening and brief interventions; TS, training and support

## Additional file

Additional file 1: Detailed description of applied implementation strategies. (PDF 971 kb)
