# Supplementary material for: Impact of primary healthcare providers’ initial role security and therapeutic commitment on implementing brief interventions in managing risky alcohol consumption: a cluster randomised factorial trial
Source: Implement Sci. 2016 Jul 16;11:96. doi: 10.1186/s13012-016-0468-5 (PMC4947288; doi:10.1186/s13012-016-0468-5)
Supplement: Additional file 1: — Detailed description of applied implementation strategies. (PDF 971 kb) [file 13012_2016_468_MOESM1_ESM.pdf]

Improving the delivery of brief interventions for heavy drinking in primary health care: outcome results of the ODHIN five country cluster randomized factorial trial

Peter Anderson[1, 2] , Preben Bendtsen[3], Fredrik Spak[4], Jillian Reynolds[5] , Colin Drummond[6,7], Lidia Segura[8], Myrna N Keurhorst[9], Jorge Palacio-Vieira[8], Marcin Wojnar[10], Kathryn Parkinson[1], Joan Colom[8], Karolina Kłoda[11], Paolo Deluca[6], Begoña Baena[8], Dorothy Newbury-Birch[1], Paul Wallace[12], Maud Heinen[9] , Amy Wolstenholme[6], Ben van Steenkiste[2], Artur Mierzecki[11], Katarzyna Okulicz-Kozaryn[13], Gaby Ronda[2], Eileen Kaner[1], Miranda GH Laurant[9, 14], Simon Coulton[15], Toni Gual[5],

[1] Institute of Health and Society, Newcastle University, Newcastle, England
[2] Department of Family Medicine, Maastricht University, Maastricht, the Netherlands.
[3] Department of Medical Specialist and Department of Medicine and Health, Linköping University, Motala, Sweden.
[4] Department of Social Medicine, University of Gothenburg, Gothenburg, Sweden.
[5] Institut Clínic de Neurosciences, Hospital Clínic de Barcelona, Barcelona, Spain.
[6]National Addiction Centre, Institute of Psychiatry, King's College London, London, England.
[7]National Institute for Health Research Biomedical Research Centre for Mental Health, South London and Maudsley NHS Foundation Trust, London, England.
[8] Program on Substance Abuse, Public Health Agency, Government of Catalonia, Barcelona, Spain.
[9] Radboud university medical center, Radboud Institute for Health Sciences, Scientific Institute for Quality of Healthcare (IQ healthcare), Nijmegen, the Netherlands.
[10] Department of Psychiatry, Medical University of Warsaw, Warsaw, Poland.
[11] Independent Laboratory of Family Physician Education, Pomeranian Medical University, Szczecin, Poland.
[12] Research Department of Primary Care and Population Health, University College London, London, England.
[13] State Agency for Prevention of Alcohol-Related Problems, Warsaw, Poland.
[14] HAN University of Applied Sciences, Faculty of Health and Social Studies, Nijmegen, the Netherlands
[15] Centre for Health Service Studies, University of Kent, Canterbury, England.

Correspondence to: Peter Anderson, Institute of Health & Society, Newcastle University, Baddiley-Clark Building, Richardson Road, Newcastle, NE2 4AX, England.
Email: Peter.Anderson@newcastle.ac.uk

Word Count: 3371

This article has been accepted for publication and undergone full peer review but has not been through the copyediting, typesetting, pagination and proofreading process which may lead to differences between this version and the Version of Record. Please cite this article as doi: 10.1111/add.13476

This article is protected by copyright. All rights reserved.

Abstract

Aim To test if training and support, financial reimbursement, and option of referring screen positive patients to an internet-based method of giving advice (eBI) can increase primary health care providers' delivery of AUDIT-C based screening and advice to heavy drinkers.

Design Cluster randomized factorial trial with 12-week implementation and measurement period.

Setting Primary health care units (PHCU) in different locations throughout Catalonia, England, Netherlands, Poland and Sweden.

Participants 120 PHCU, 24 in each of Catalonia, England, Netherlands, Poland and Sweden**.**

Interventions PHCUs were randomized to one of eight groups: care as usual, training and support (TS), financial reimbursement (FR), and eBI; paired combinations of TS, FR and eBI, and all of FR, TS and eBI.

Measurements The primary outcome measure was the proportion of eligible adult (age 18+ years) patients screened during a 12-week implementation period. Secondary outcome measures were proportion of screen positive patients advised; and, proportion of consulting adult patients given an intervention (screening and advice to screen positives) during the same 12-week implementation period.

Findings During a 4-week baseline measurement period, 5.9 (95% CI 3.4 to 8.4) per 100 adult patients consulting per PHCU were screened for their alcohol consumption. Based on the factorial design, the ratio of the logged proportion screened during the 12-week implementation period was 1.48 (95% CI 1.13 to 1.95) in PHCU that received TS versus PHCU that did not receive TS; for FR, the ratio was2.00 (95% CI 1.56 to 2.56). The option of referral to eBI did not lead to a higher proportion of patients screened. The ratio for TS plus FR was2.34 (95% CI 1.77 to 3.10), and the ratio for TS plus FR plus eBI was1.68 (95% CI 1.11 to 2.53).

Conclusions Providing primary health care units with training, support and financial reimbursement for delivering AUDIT-C based screening and advice to heavy drinkers increases screening for alcohol consumption. Providing primary health care units with the option of referring screen positive patients to an internet-based method of giving advice does not appear to increase screening for alcohol consumption.

Trial registration ClinicalTrials.gov. Trial identifier: NCT01501552

This article is protected by copyright. All rights reserved.

Introduction

Alcohol consumption is a wholly or contributory cause for more than 200 diseases, injuries and other health conditions with three digit ICD-10 codes [1]. The cardio-protective effect of low-risk patterns of alcohol consumption disappears in the presence of heavy episodic drinking [2]. Globally, alcohol is the sixth most important risk factor for ill-health and premature death [3]. Reduction in alcohol consumption is essential to achieve global targets of reducing deaths from non-communicable diseases by 25% between 2010 and 2025 [4]. Heavy drinkers who reduce their drinking reduce their risk of mortality in comparison to those who continue heavy drinking [5-6]. The higher the level of drinking, the stronger the effects of a given reduction [7]. Systematic reviews demonstrate that primary health care based screening and brief advice programmes are effective in reducing alcohol consumption and related harm, with the evidence strongest for adults, and less so for adolescents [8-10].

Many national and international guidelines recommend routine screening in primary health care and the offer of advice to screen positive patients (e.g. [11-13]). However, in many jurisdictions there is a large gap between need and provision of advice. Elsewhere, we have shown that only 5.3% of eligible patients consulting their primary health care provider over a four-week period were screened for their alcohol consumption (average across Catalonia, England, Netherlands, Poland and Sweden) [14]. It is possible to increase the proportion of eligible patients screened. A meta-regression analysis of 29 studies found that professional and patient-oriented implementation strategies could improve screening (standardized effect 0.53;95%-CI (confidence interval) 0.28 to 0.78) and advice (standardized effect 0.64;95%-CI 0.27 to 1.02) rates [15].

In this paper, we report on a five country study that tested the hypothesis that the provision of each of training and support, financial reimbursement, and the option of referring identified heavy drinking patients to an internet based method of delivering advice (eBI) [16], singly and in combination to primary health care providers, will increase the proportion of adult patients (aged 18+ years) screened and given brief advice, compared to no provision. In our study, the unit of randomization, intervention, and analysis is the primary health care unit (PHCU) and not the individual primary health care provider working within a PHCU.

Methods

Design

In a cluster randomized 2x2x2 factorial trial [for trial protocol, see 17 – there were no trial deviations], the impact of the three different implementation strategies on screening and advice for heavy drinking operationalized by AUDIT-C [see 18] was studied (Fig. 1). Data were collected between August 2012 and December 2013.

Fig.1. Trial Flow chart.

Participants

PHCUs with approximately 5,000-20,000 registered patients from five jurisdictions (Catalonia, England, Netherlands, Poland and Sweden) were the unit of randomization and implementation.

This article is protected by copyright. All rights reserved.

PHCUs who agreed to participate in the study were volunteers drawn from administrative or academic registries of PHCUs at national or regional levels. Eligible providers in each PHCU included any fully trained full or part-time medical practitioner, nurse or PHCU assistant with a permanent appointment working in the PHCU. Not all providers within each PHCU participated in the study.

Implementation strategies

PHCU were recruited between March and July 2013. After formal agreement of the PHCU to take part in the trial, a 4-week baseline measurement period took place. After a 2-6-week gap, the 12-week implementation period occurred, with the start date for each country between November 2012 and May 2013. All seven groups received the same input as controls but with additional components added.

1. Control Group: The control group was given a package containing a summary card of the national guideline recommendations for screening and advice for hazardous and harmful alcohol consumption, without demonstration. In Poland, the card was adapted from the PHEPA guidelines [19-20]. Instructions were given on how to complete the trial record sheet, and providers were asked to screen all adult patients (aged 18+ years) with AUDIT-C.

2. Training and support (TS): In addition to receiving the same package as the control group, the TS group was offered two initial 1-2 hours face-to-face educational trainings, and one (10-30 minutes) telephone support call to the lead PHCU contact person during the 12-week implementation period. Each country used an adapted existing country-based TS package. In Poland, the TS package was based on the PHEPA training programme [21].

3. Financial reimbursement (FR): The financial reimbursement group was paid for screening and advice activities during the 12-week implementation period. In Catalonia, a maximum ceiling rate of €250 per provider was established, and fees were calculated based on the average individual performance of the 12-week implementation period. In England, fees were €6 per screening and €25 per advice, with a maximum ceiling rate of €2200 per PHCU. In the Netherlands, fees were €9 per screening and €13.50 per advice, with a maximum ceiling rate of €1250 per PHCU. In Poland, fees were €1.25 per screening and €10 per advice, with no ceiling rate. In Sweden, fees were €2 per screening and €15 per advice with a maximum ceiling rate of €3300 per PHCU.

4. eBI: In addition to receiving the same package as the control group, the eBI group was asked to refer identified at risk patients with an e-leaflet to an approved eBI specific package, which was country specific, or, for Poland based on the WHO e-SBI programme.

5. TS and financial reimbursement (TSFR): The TS and FR group received the control group package, training and support, and the financial reimbursement as described above.

6. TS and eBI (TSeBI): The TS and eBI group received the control group package, training and support as above, and were asked to refer identified at risk patients to eBI as above.

7. Financial reimbursement and eBI (FReBI): The FR and eBI group received the control group package, were asked to refer identified at risk patients to eBI, and received the same financial reimbursement as for FR, even if providers only referred screen-positive patients to eBI.

8. TS, financial reimbursement and eBI (TSFReBI: The TS, FR and eBI group received the control group package and training and support as above. They were asked to refer identified at risk patients to eBI and received financial reimbursement as described in group 7 above.

This article is protected by copyright. All rights reserved.

PHCUs were asked to screen all adult patients (aged 18+ years) who consulted the PHCU using a paper version of AUDIT-C, except in Catalonia, where a computerized version was used. Screen positives were defined in Catalonia and England as men and women who scored ≥5 on AUDIT-C, and in Poland, Netherlands and Sweden as men who scored ≥5 and women who scored ≥4 on AUDIT-C. PHCU were asked to deliver brief advice of 5-15 minutes duration to screen positives, with the length and format of the advice based on country specific guidelines or, for Poland, the European guidelines developed by PHEPA [19]. Providers who were allocated to eBI activity were asked to refer screen-positive patients to a computerized advice programme, taking a few minutes to explain why the patient ought to log on to the site.

Outcomes

Screening and brief advice activity was measured at two time points: during the 4-week baseline period, and during the 12-week implementation period, using paper tally sheets completed by the providers, with the exception of Catalonia, where electronic patient records were completed by the providers. The tally sheets included AUDIT-C questions, AUDIT-C scores, and tick boxes to indicate the type of advice (oral advice, an advice leaflet, referral to the eBI programme, or referral for advice to another provider in or outside the PHCU) that was delivered. For the one PHCU that dropped out of the study after the baseline measurement, data outcome measurements during the 12-week implementation period were set as the rates for the baseline measurement period (intention to treat analysis).

The primary outcome measure was the proportion of eligible patients screened, the number of patients screened divided by the number of adult consultations of the participating providers per PHCU. The secondary outcome measures were the **proportion screen positives advised**, the number of AUDIT-C positive patients that received one or more of oral advice, an advice leaflet, referral to the eBI programme, or referral for advice to another provider in or outside the PHCU, divided by the total number of screen positive patients per PHCU; and the **proportion of consulting adult patients intervened (screening and advice to screen positives)**, the number of AUDIT-C positive patients that received one or more of oral advice, an advice leaflet, referral to the eBI programme, or referral for advice to another provider in or outside the PHCU, divided by the total number of adult consultations of the participating providers per PHCU.

As distributional assumptions of the outcome measures were violated, a natural logarithm transformation was undertaken. As this approach creates some issues with outcomes with a zero value, 0.001 was added to each proportion. At baseline, 7/124 PHCU returned a zero proportion of patients screened, and 5/124 PHCU returned a zero proportion of screen-positive patients advised. During the 12-week implementation period, no PHCU returned a zero proportion of patients screened, and 3/124 PHCU returned a zero proportion of screen-positive patients advised. In order to test the validity of this assumption a sensitivity analysis was undertaken using the exact proportions but excluding those PHCU with an outcome of zero.

Randomization and blinding

Randomization of the PHCU took place after formal agreement of the PHCU to take part in the trial. The PHCUs were randomly allocated to one of the eight groups by the ODHIN coordinating centre in Barcelona, using Microsoft Office Excel computerized randomization. Stratified randomization by country was used to ensure equal numbers of 15 PHCUs per eight allocation group, with equal numbers per allocation group per country (three PHCU per allocation group per country). The

This article is protected by copyright. All rights reserved.

country-based research teams were informed of the allocation after collection of the baseline measurement. The PHCU were informed by the country-based research teams of the allocation after collection of the baseline measurement.

Sample size

It was estimated that 56 PHCUs (seven per eight allocation groups) with a minimum of 1,000 adult patients per month would be needed for a 80% chance of detecting an increase in the proportion of patients screened from 8% to at least 12% (ICC (Intraclass Correlation Coefficient) = 0.029) and that 120 PHCUs (15 per eight allocation groups) would be needed for a 80% chance of detecting an increase in the proportion of consulting adult patients given an intervention from 4% to at least 6% (ICC = 0.029) (alpha = 0.05). As country was used as stratification criteria each country included a minimum of 24 PHCU [17, data based on 22].

Statistical methods

The primary outcome for the study was the proportion screened over the 12-week implementation period and this was analysed by allocated group. Distributional assumptions were assessed and transformations were undertaken where appropriate. The analysis was conducted on an intention to treat basis. The primary outcome was analysed as a generalised linear model with proportion screened in the 4-week baseline period incorporated as a covariate. As the study is hierarchical in nature with PHCU nested within country, a multi-level approach was employed using country with random intercepts and slopes. Means by group and 95% confidence intervals (95% CI) are presented for each allocated group. Mean differences and 95% CI are presented with reference to the control group. In order to aid interpretation, the ratio of proportion screened in the allocated group versus the control group and associated 95% CI are also presented and an overall level of significance presented.

As the study was conceived as a factorial design, a second analysis explored individual, TS, FR, EBI, and combined, TSFR, TSEBI, FREBI, TSFREBI factors of the intervention (For examples of factorial designs, see [23-25]). These were analysed in a similar manner with an exploration for potential interactions using a step-up approach and interpreted using the r-square statistic. Where interactions were identified they were incorporated into the regression equation. Additional secondary analysis was conducted on the proportion of screen positives who were provided with advice and the proportion of all patients who consulted who were screened, and if screen positive, given advice. Results are presented in a similar manner to the primary outcome. Analysis was conducted using Stata and MLWin.

RESULTS

In total, 618 PHCU were approached across the five countries to reach the required sample size of 120 PHCU's, 24 per country. The overall enrolment rate of 19.4% varied across countries: Catalonia 65%, England 7%, Netherlands 7%, Poland 46%, Sweden 24%. The mean number of patients registered in each PHCU was 10,000. There was a mean of 1500 adult (age 18+ years) consultations per PHCU during the 4-week baseline period, mean age 53 years (SD=6), of whom 55% were men. Just over half of the participating providers per PHCU were doctors (55%), 38% nurses, and 7% practice assistants. The mean age of the participating providers per PHCU was 47 years (SD=5), and 74% were women.

This article is protected by copyright. All rights reserved.

During the 4-week baseline period, the mean proportion of eligible patients screened per PHCU was 0.059 (95%CI 0.034 to 0.084); the mean proportion of screen positives advised per PHCU was 0.737 (95% CI 0.606 to 0.868); and the mean proportion consulting adult patients given an intervention per PHCU was 0.011 (95%CI 0.005 to 0.017). Table 1 shows the mean and standard error of proportion screened, advised and intervened over the 4-week baseline and 12-week implementation periods. Table 2 illustrates that the proportion of patients screened, advised and intervened generally dropped off for most allocation groups during the 12-week implementation period.

Table 3 shows the mean proportion screened during the 12-week implementation period by allocated group. Significant effects were observed for the TS, TSFR and TSFREBI groups compared to the control group and in all cases the effect favoured the intervention group over the control. Table 4 shows that a similar effect was observed for these groups for the proportion of screen positives receiving advice but no significant effects were observed for the proportion receiving an intervention (proportion of consulting adult patients given an intervention (screening and advice to screen positives)).

Discussion

Overall findings

During a 4-week baseline measurement period, screening for alcohol consumption was delivered by primary health care providers to just under 6% of adult consultations, with 74% of screen positives given advice. Overall, brief advice for AUDIT-C screen positive patients was delivered by primary health care providers to 11 per 1,000 adult consultations. An AUDIT-C cut-off score of 5 is equivalent to a consumption level of about 20 grams of alcohol per day [26]. Amongst EU citizens aged 15-64 years, 230/1,000 women regularly drink 20 grams of alcohol or more per day and 300/1, 000 men regularly drink 40 grams of alcohol or more per day [27]. Of those screened in the ODHIN study, 330/1000 were AUDIT-C positive; this suggests that only some 3% of those who might benefit from brief advice were receiving it.

We found the provision of training and support and of financial reimbursement led to a higher proportion of consulting adult patients being screened for alcohol consumption. The offer of eBI referral did not impact this proportion. We consider the lack of an impact of eBI due to a lack of familiarization by the providers of eBI, and perhaps lack of trust in its impact. We did not find evidence of extra synergy from combined strategies, compared with single strategies alone.

The proportion of screen positive patients given brief advice was very high at baseline (74%). This is likely to explain our inability to demonstrate an impact of training and support and financial reimbursement in changing the proportion of screen positive patients given brief advice.

Strengths and weaknesses

One strength of the present study is its factorial design, which ensured that it had sufficient power to detect small changes with a relatively small number of PHCU (120). Another strength of the study is that it was conducted across five different European jurisdictions, with differing health system financing and management structures.

This article is protected by copyright. All rights reserved.

One weakness of the study is that the involved PHCUs were volunteers drawn from administrative or academic registries until the required sample size was achieved. Data is not available to indicate whether or not the volunteer PHCUs were representative of PHCUs in the five countries as a whole. However, we do know that general practitioners in the present study had higher role security (mean 21.59, SD 3.20) when working with drinkers than representative samples of general practitioners from the same countries (mean 20.55, SD 2.94), anova, F=25.4, p<0.001; and, had higher therapeutic commitment when working with drinkers (mean 26.76, SD 4.54) than the representative sample (mean 24.67, SD 4.74), anova, F=57.75, p<0.001 [14]. Role security and therapeutic commitment are measures of attitudes toward working with drinkers, and thus the volunteer PHCUs seem more motivated to working with drinkers than PHCUs from the same country in general.

A second weakness of the present study was that the outcome measures were of provider behaviour, rather than patient outcomes. Another weakness of the study is that the record sheet to measure AUDIT-C included the options for giving advice. In itself, this is an organizational intervention to support provider behaviour that, whilst equal across all intervention groups, probably led to the high advice rates for positive screens (74%). Completion of the record sheet was made by the provider, and the study had no independent check that the advice was actually carried out, or that a screen or advice were done without being registered on the record sheet. Another weakness of the study is the short time span of the implementation period. Resourcing of the study constrained the implementation and analysis period to twelve weeks. During this twelve weeks, as indicated by table 2, the proportion of patients screened, advised and intervened tended to drop off in most allocation groups, suggesting that the impact of the implementation strategies might be quite short-lived. Our study was restricted to adults (aged 18+ years), as the evidence for the effectiveness of primary health care delivered brief advice is much stronger for adults than for adolescents. Thus, we do not know if our interventions would have increased the proportion of adolescents screened and advised.

Comparison with other studies

The impact of training and support is similar to the results of the World Health Organization four country (Australia, Belgium, Catalonia and England) collaborative randomized controlled trial which demonstrated the effectiveness of training and support in promoting screening and Intervention for hazardous and harmful alcohol consumption [22, 28]. In the WHO study, the odds ratios for the impact of high training and support on increasing higher screening proportions (defined as 20% or more) was 2.2 (95% CI=1.3 to 3.1) and on increasing higher intervention proportions (defined as 10% or more) was 2.8 (95% CI = 1.6 to 4.0) [32].

In contrast, a cluster randomized controlled trial in the Netherlands, which investigated the impact of an improvement programme combining professional, organisation, and patient directed activities, failed to find an impact of the intervention on the number of adult patients who received screening and advice [29]. One of the given reasons for failing to find an impact was sub-optimal implementation of the programme due to difficulties in recruiting GPs and in motivating GPs for participation in the tailored parts of the programme.

Implications for service commissioners and policy makers

The potential of screening and brief advice programmes to improve health (and sometimes to reduce costs) has been shown elsewhere [30-31]. With strong government support for alcohol brief interventions, reinforced by financial and performance management arrangements, guidance and

This article is protected by copyright. All rights reserved.

strategic leadership, as well as training, it is possible to increase alcohol screening and brief interventions (see [13, 32-34]).

We included the option of referral to an eBI programme as one of the implementation strategies in the belief that this might encourage higher screening activity, as providers did not then have to deliver a brief advice themselves. The failure of this strategy to impact on any of the outcomes would suggest that providers in this study are not yet ready to refer patients to eBI programmes. Elsewhere, we have shown that providers who more strongly believe that heavy drinking is the drinker's own responsibility report that they are less likely to engage in delivering brief advice [35]. Thus, for the time being, it might be preferable to market eBI programmes directly to drinkers, rather than through their primary health care providers, whilst more studies are undertaken to explore how referral to eBI could be best organized and implemented.

Based on the ODHIN findings, we would recommend that all jurisdictions could consider providing support for alcohol brief advice based on training and guidance, financial and performance management arrangements, and strategic leadership, so as to increase the volume of brief interventions delivered to heavy drinking patients in primary health care.

Consort statement

This paper adheres to the CONSORT 2010 guidelines [36].

Acknowledgements

We thank all participating PHCUs and practitioners for their support with the trial. We thank Steven Teerenstra (Statistician, at the department Health Evidence Radboud university medical centre) who assisted PA, MK and ML in designing the analysis plan for factorial RCT design. We thank Birgit Jansen (research assistant, Scientific Institute for Quality of Healthcare (IQ healthcare), Radboud university medical center) and Paddy Hinsssen (research assistant, Faculty of Health, Medicine and Life Sciences, Maastricht University), for executing many logistic procedures to gather the tally sheets. We thank the Catalan society of Family and Community Medicine, the Catalan Association of Family and Community Nursing and the Alcohol Network of Referents on Alcohol (XaROH) of the Programa Beveu Menys, who collaborated in the implementation of the trial in Catalonia. We thank Sebastián Calero from the Catalan Health Institute's Healthcare Issues' Area of Clinical Development, Carmen Olmos and Manuel Iglesias from the eCAP Functional Competences Centre and Manuel Medina, Francesc Fina, Leonardo Méndez and Eduard Hermosilla from the Information system for research development in primary Health care (SIDIAP database) for supporting the Catalan ODHIN team in the adaptation of the electronic medical records (eCAP) and in the data extraction for the trial. The following individuals were part of the WP5 ODHIN group, besides the authors of this manuscript: Trudy van der Weijden (Netherlands) and Michel Wensing (Netherlands).

Contributions

All authors, except PA, were involved in implementing the trial in their jurisdictions. SC and PA undertook the analyses. PA wrote the drafts of the manuscript. All other authors revised the manuscript critically. All authors read and approved the final manuscript.

This article is protected by copyright. All rights reserved.

Funding

The research leading to these results or outcomes has received funding from the European Union's Seventh Framework Programme for research, technological development and demonstration under grant agreement no 259268 – Optimizing delivery of health care intervention (ODHIN). Participant organisations in ODHIN can be seen at: www.odhinproject.eu. Radboud university medical center received co-funding from The Netherlands Organisation for Health Research and Development (ZonMW, Prevention Program), under Grant Agreement nº 200310017 – ODHIN – Optimizing delivery of healthcare interventions in the Netherlands, according Art.II.17 of the FP7 EC Grant Agreement. Colin Drummond is partly funded by the NIHR Biomedical Research Centre for Mental Health at South London and Maudsley NHS Foundation Trust and King's College London and partly funded by the NIHR Collaborations for Leadership in Applied Health Research and Care South London at King's College Hospital NHS Foundation Trust. The views expressed are those of the author(s) and not necessarily those of the NHS, the NIHR, the Department of Health, or the European Commission. Pomeranian Medical University in Szczecin received co-funding for the ODHIN project from the Polish science financial resources in the years 2012-2014.

Competing interests

All authors have completed the ICMJE uniform disclosure form at www.icmje.org/coi_disclosure.pdf (available on request from the corresponding author) and declare: no financial relationships with any organisations that might have an interest in the submitted work in the previous three years; no other relationships or activities that could appear to have influenced the submitted work; with the following exceptions: Dr. Gual reports grants and personal fees from Lundbeck, and D&A Pharma, grants from Teva, and personal fees from Abbivie, outside the submitted work. Professor Wallace reports personal fees from Lundbeck, Drinkaware and the Safe Sensible London Partnership. These declarations do not alter our adherence to PLOS ONE policies on sharing data and materials

References

1. World Health Organization. Global status report on alcohol and health 2014. Geneva: World Health Organization, 2014.
2. Roerecke M, Rehm J. Irregular heavy drinking occasions and risk of ischemic heart disease: A systematic review and meta-analysis. Am J Epidemiol. 2010 171:633–44. doi: 10.1093/aje/kwp451.
3. GBD 2013 Risk Factors Collaborators. Global, regional, and national comparative risk assessment of 79 behavioural, environmental and occupational, and metabolic risks or clusters of risks in 188 countries, 1990-2013: a systematic analysis for the Global Burden of Disease Study 2013. *Lancet*. 2015; 386:2287-323..
4. Kontis V, Mathers, CD, Rehm J, Stevens G A, Shield K D, Bonita R, et al. Contribution of six risk factors to achieving the "25×25" NCD mortality reduction target. Lancet 2014 http://dx.doi.org/10.1016/ S0140-6736(14)60616-4.

This article is protected by copyright. All rights reserved.

5. Emberson JR, Shaper AG, Wannamethee SG et al. Alcohol intake in middle age and risk of cardiovascular disease and mortality: accounting for intake variation over time. Am J Epidemiol 2005 161:856–63.

6. Fillmore KM, Kerr WC, Bostrom A. Changes in drinking status, serious illness and mortality. J Stud Alcohol 2003 64:278–85.

7. Rehm J, Roerecke, M. Reduction of Drinking in Problem Drinkers and All-Cause Mortality. Alcohol and Alcoholism 2013 Vol. 48, No. 4, pp. 509–513.

8. Kaner, EF, Beyer F, Dickinson H, Pienaar E, F FC, Schlesinger C, Heather N, et al. Effectiveness of brief alcohol interventions in primary care populations. *Cochrane Database Syst Rev* 2007, 18(2):CD004148.

9. Jonas DE, Garbutt JC, Amick HR, Brown JM, Brownley KA, Council CL, et al. Behavioral counseling after screening for alcohol misuse in primary care: a systematic review and meta-analysis for the U.S. Preventive Services Task Force. Ann Intern Med. 2012;157:645-54. [PMID: 23007881]

10. O'Donnell A, Anderson P, Newbury-Birch D, Schulte B, Schmidt C, Reimer J, et al. The impact of brief alcohol interventions in primary healthcare: A systematic review of reviews Alcohol and Alcoholism. 2014;49(1):66-78.

11. National Institute for Health and Clinical Excellence. Alcohol-use disorders - preventing the development of hazardous and harmful drinking. http://guidanceniceorguk/PH24. 2010.

12. Dua T, Barbui C, Clark N, Fleischmann A, Poznyak V, van Ommeren, M et al. Evidence based guidelines for mental, neurological and substance use disorders in low- and middle-income countries: Summary of WHO recommendations. PLOS Medicine, 2011 8 (11), 1-11.

13. Health Scotland (2013). Alcohol Advice 2012/2013. https://isdscotland.scot.nhs.uk/Health-Topics/Drugs-and-Alcohol-Misuse/Publications/2013-06-25/2013-06-25-ABI-Report.pdf?89017885924

14. Bendsten, P, Anderson P, Wojnar M, Newbury-Birch D, Müssener, U et al. professional's attitudes do not influence screening and brief intervention rates for hazardous and harmful drinkers: results from ODHIN study. Alcohol and Alcoholism 2015 doi: 10.1093/alcalc/agv020

15. Keurhorst M, van de Glind I, Bitarello do Amaral-Sabadini M, Anderson P, Kaner E, Newbury-Birch D et al. Determinants of successful implementation of screening and Advice for hazardous and harmful alcohol consumption in primary healthcare. A systematic review and meta-regression analysis. Addiction 2015 110:877–900.

16. Donoghue K, Patton R, Phillips T, Deluca P, Drummond C. The Effectiveness of Electronic Screening and Intervention for Reducing Levels of Alcohol Consumption: A Systematic Review and Meta-Analysis. *(J Med Internet Res 2014;16(6):e142)* doi:10.2196/jmir.3193.

17. Keurhorst M, Anderson P, Spak F, Bendtsen P, Segura L, Colom J et al. Implementing training and support, financial reimbursement, and referral to an internet-based Advice program to improve the early identification of hazardous and harmful alcohol consumption in primary care (ODHIN): study protocol for a cluster randomized factorial trial. Implementation Science 2013 8:11. doi:10.1186/1748-5908-8-11.

18. Jonas DE, Garbutt JC, Brown JM, Amick HR, Brownley KA, Council CL, et al. Screening, Behavioral Counseling, and Referral in Primary Care to Reduce Alcohol Misuse. Comparative Effectiveness Review No. 64. Rockville, MD: Agency for Healthcare Research and Quality; July 2012. Accessed at www.ncbi .nlm.nih.gov/books/NBK99199/ on 16 April 2013.

This article is protected by copyright. All rights reserved.

19. Anderson P, Gual A, Colom J. Alcohol and Primary Health Care: Clinical Guidelines on Identification and Advice In. Barcelona: Department of Health of the Government of Catalonia; 2005.

20. Fudała J. Rozpoznawanie problemów alkoholowych i krótkie interwencje (*Recognition of Alcohol Problems and brie interventions*). Shortened version of the: Anderson P, Gual A, Colom J: Alcohol and Primary Health Care: Clinical Guidelines on Identification and Interventions In. Barcelona: Department of Health of the Government of Catalonia; 2005., PARPA, Warsaw, 2009

21. Gual A, Anderson P, Segura L, Colom J. Alcohol and primary health care: Training programme on identification and Advice. Barcelona: department of Health of the Government of Catalonia, 2005.

22. Funk M, Wutzke S, Kaner E, Anderson P, Pas L, McCormick R et al. A multi country controlled trial of strategies to promote dissemination and implementation of brief alcohol intervention in primary health care: Findings of a WHO Collaborative Study. Journal of Studies on Alcohol 2005 66 379-388.

23. Day L, Fildes B, Gordon I, Fitzharris M, Flamer H, Lord S. Randomised factorial trial of falls prevention among older people living in their own homes. BMJ 2002; 325:128.

24. Sedgwick P. Randomised controlled trials with full factorial designs. BMJ 2012; 345:e5114.

25. Montgomery AA, Peters TJ, Little P. Design, analysis and presentation of factorial randomised controlled trials. BMC Medical Research Methodology. 2003;3:

26. Rubinsky AD, Dawson DA, Williams EC, Kivlahan DR Bradley KA AUDIT-C Scores as a Scaled Marker of Mean Daily Drinking, Alcohol Use Disorder Severity, and Probability of Alcohol Dependence in a U.S. General Population Sample of Drinkers. Alcoholism: Clinical and Experimental Research 2013 37 1380-1390.

27. Rehm J, Shield KD, Rehm MX, Gmel G, Frick, U. Alcohol consumption, alcohol dependence, and attributable burden of disease in Europe: potential gains from effective interventions for alcohol dependence. Toronto, Canada: Centre for Addiction and Mental Health, 2012.

28. Anderson P, Kaner E, Wutzke S, Funk M, Heather N, Wensing M et al. Attitudes and managing alcohol problems in general practice: an interaction analysis based on Findings from a WHO Collaborative Study. Alcohol and Alcoholism 2004 39:351-356.

29. van Beurden I, Anderson P, Akkermans RP, Grol RP, Wensing M, Laurant MG. Involvement of general practitioners in managing alcohol problems: a randomised controlled trial of a tailored improvement programme. Addiction 2012, 107:1601–1611.

30. Purshouse, R, Brennan, A, Rafia, R Modelling the Cost-Effectiveness of Alcohol Screening and Advice in Primary Care in England. *Alcohol Alcohol* 2013 **48**, 180-188.

31. Angus C. Cost-effectiveness of a programme of screening and Advice for alcohol in primary care in Italy. BMC Family PHCU 2014 15:26. doi:10.1186/1471-2296-15-26

32. Health Scotland (2008). H4 ABI: Target and Background Information. http://www.healthscotland.com/documents/3201.aspx.

33. Health Scotland (2011). An evaluation to assess the implementation of NHS delivered Alcohol Brief Interventions: Final Report September 2011. http://www.healthscotland.com/documents/5438.aspx

34. Health Scotland (2011). HEAT Standard: Alcohol Brief Interventions. National Guidance : 2012-13 http://www.healthscotland.com/uploads/documents/17579-ABI%20HEAT%20Standard%20National%20Guidance%202012%20-13%20-%20PDF.pdf

This article is protected by copyright. All rights reserved.

35. Anderson P, Wojnar J, Jakubczyk A, Gual A, Reynolds J, Segura L et al. Managing Alcohol Problems in General Practice in Europe: Results from the European ODHIN Survey of General Practitioners. Alcohol and Alcoholism Vol. 49, No. 5, pp. 531–539.

36. Moher D, Hopewell S Schulz KF CONSORT 2010 Explanation and Elaboration: updated guidelines for reporting parallel group randomised trials. *BMJ* 2010;340:c869 doi: 10.1136/bmj.c869

This article is protected by copyright. All rights reserved.

Table 1: Mean proportion and standard error of those screened, advised and intervened during 4-week baseline period and 12-week implementation period by allocated group.

	Mean proportion (SE)*	
	**Baseline**	**12-week implementation**
**Screen**		
Control	0.0681 (0.0212)	0.0349 (0.0064)
TS	0.0676 (0.0125)	0.0548 (0.0122)
FR	0.0667 (0.0136)	0.1182 (0.0322)
EBI	0.0970 (0.0231)	0.0574 (0.0139)
TS+FR	0.0524 (0.0095)	0.1758 (0.0557)
TS+EBI	0.0452 (0.0124)	0.0367 (0.0106)
FR+EBI	0.0587 (0.0183)	0.0659 (0.0188)
TS+FR+EBI	0.0568 (0.0141)	0.0786 (0.0151)
**Advice**		
Control	0.7961 (0.0517)	0.6270 (0.0820)
TS	0.7921 (0.0712)	0.8743 (0.0364)
FR	0.6824 (0.0796)	0.8288 (0.0516)
EBI	0.6925 (0.0802)	0.8062 (0.0596)
TS+FR	0.7520 (0.0943)	0.9048 (0.0356)
TS+EBI	0.7728 (0.0617)	0.7960 (0.0926)
FR+EBI	0.7564 (0.0964)	0.7627 (0.0790)
TS+FR+EBI	0.6628 (0.0661)	0.8626 (0.0304)
**Intervene**		
Control	0.0134 (0.0035)	0.0056 (0.0013)
TS	0.0149 (0.0044)	0.0108 (0.0035)
FR	0.0097 (0.0018)	0.0180 (0.0044)
EBI	0.0167 (0.0035)	0.0098 (0.0021)
TS+FR	0.0125 (0.0033)	0.0346 (0.0098)
TS+EBI	0.0128 (0.0028)	0.0091 (0.0022)
FR+EBI	0.0121 (0.0039)	0.0078 (0.0018)
TS+FR+EBI	0.0071 (0.0017)	0.0154 (0.0029)

* N=15 for all groups, with the following exceptions:
Screen, 12-week, FR=14 (one PHCU dropped out)
Advice, baseline, TSeBI=11; Control, TSFR=13; TS, eBI, FReBI, TSFReBI=14 (when proportion screened=0, proportion advised cannot be calculated)
Advice, 12-week, FR=14 (one PHCU dropped out)
Intervene, 12-week, FR=14 (one PHCU dropped out)

This article is protected by copyright. All rights reserved.

Table 2: Mean proportion and standard error of those screened, advised and intervened during weeks 1-4, 5-8 and 9-12 of 12-week implementation period by allocated group.

		Mean proportion (SE)		
		Weeks of 12-week implementation period		
		1-4	5-8	9-12
**Screen**				
	Control	0.0388 (0.00716)	0.0344 (0.00720)	0.0256 (0.00573)
	TS	0.0640 (0.01409)	0.0553 (0.01338)	0.0491 (0.00989)
	FR	0.1428 (0.04558)	0.1119 (0.03378)	0.0999 (0.02403)
	EBI	0.0718 (0.01931)	0.0574 (0.01045)	0.0515 (0.01405)
	TS+FR	0.1905 (0.05974)	0.1541 (0.04246)	0.1503 (0.04557)
	TS+eBI	0.0493 (0.01126)	0.0406 (0.01042)	0.0371 (0.00771)
	FR+eBI	0.1035 (0.02756)	0.0504 (0.01310)	0.0521 (0.01791)
	TS+FR+eBI	0.1390 (0.04531)	0.0761 (0.01413)	0.0697 (0.01339)
**Advice**				
	Control	0.7147 (0.08130)	0.7124 (0.08246)	0.5851 (0.10382)
	TS	0.8850 (0.03129)	0.8379 (0.04846)	0.8797 (0.05458)
	FR	0.8610 (0.03788)	0.8692 (0.05882)	0.7561 (0.07170)
	EBI	0.7886 (0.06196)	0.8188 (0.06933)	0.8046 (0.06362)
	TS+FR	0.9253 (0.02497)	0.8925 (0.03735)	0.8603 (0.06547)
	TS+eBI	0.7940 (0.08886)	0.8239 (0.07955)	0.8891 (0.04522)
	FR+eBI	0.7786 (0.07417)	0.7686 (0.08424)	0.8404 (0.06984)
	TS+FR+eBI	0.8747 (0.03319)	0.8796 (0.03597)	0.8466 (0.03991)
**Intervene**				
	Control	0.0071 (0.00164)	0.0056 (0.00107)	0.0040 (0.00141)
	TS	0.0134 (0.00322)	0.0125 (0.00459)	0.0100 (0.00325)
	FR	0.0184 (0.00440)	0.0177 (0.00473)	0.0178 (0.00514)
	EBI	0.0115 (0.00283)	0.0087 (0.00139)	0.0093 (0.00247)
	TS+FR	0.0384 (0.01158)	0.0304 (0.00765)	0.0274 (0.00720)
	TS+eBI	0.0118 (0.00297)	0.0080 (0.00182)	0.0084 (0.00219)
	FR+eBI	0.0134 (0.00332)	0.0063 (0.00125)	0.0060 (0.00180)
	TS+FR+eBI	0.0246 (0.00547)	0.0142 (0.00312)	0.0103 (0.00203)

This article is protected by copyright. All rights reserved.

Table 3: Mean natural log of proportion screened during 12-week implementation period, mean difference versus control (95% CI), ratio versus control (95% CI) and significance value by allocated group.

	Mean during 12-week implementation period (95% CI)	Difference versus control (95% CI)	Ratio versus control (95% CI)	Sig.
Control	-3.589 (-4.493; -2.686)	-	1.00	-
TS	-3.233 (-4.136; -2.330)	0.357 (-0.098; 0.812)	1.43 (0.37; 2.25)	0.123
FR	-2.732 (-3.636; -1.827)	0.858 (0.392; 1.324)	2.36 (1.48; 3.76)	<0.001
EBI	-3.389 (-4.292; -2.485)	0.201 (-0.260; 0.662)	1.22 (0.77; 1.94)	0.390
TS+FR	-2.238 (-3.141; -1.335)	1.351 (0.897; 1.805)	3.86 (2.45; 6.08)	<0.001
TS+EBI	-3.379 (-4.283; -2.475)	0.210 (-0.245; 0.666)	1.23 (0.78; 1.95)	0.362
FR+EBI	-3.248 (-4.151; -2.345)	0.342 (-0.112; 0.795)	1.41 (0.89; 2.21)	0.139
TS+FR+EBI	-2.604 (-3.507; -1.701)	0.986 (0.532; 1.439)	2.68 (1.70; 4.22)	<0.001

This article is protected by copyright. All rights reserved.

Table 4: Mean natural log of proportion advised and given intervention during 12-week implementation period, mean difference versus control (95% CI), ratio versus control (95% CI) and significance value by allocated group.

	Mean during 12-week implementation period (95% CI)	Difference versus control (95% CI)	Ratio versus control (95% CI)	Sig.
**Advice**				
Control	-5.362 (-6.010; -4.714)	-	1.00	-
TS	-4.773 (-5.422; -4.125)	0.588 (0.149; 1.028)	1.80 (1.16; 2.80)	0.009
FR	-4.453 (-5.104; -3.802)	0.909 (0.461; 1.356)	2.48 (1.59; 3.88)	<0.001
EBI	-4.877 (-5.526; -4.228)	0.485 (0.044; 0.925)	1.62 (1.04; 2.52)	0.031
TS+FR	-3.808 (-4.456; -3.160)	1.554 (1.114; 1.994)	4.73 (3.05; 7.34)	<0.001
TS+EBI	-4.926 (-5.574; -4.227)	0.436 (-0.004; 0.876)	1.55 (0.99; 2.40)	0.052
FR+EBI	-4.887 (-5.536; -4.239)	0.474 (0.034; 0.915)	1.61 (1.03; 2.50)	0.035
TS+FR+EBI	-4.183 (-4.813; -3.534)	1.179 (0.738; 1.620)	3.25 (2.09; 5.05)	<0.001
**Intervention**				
Control	-0.951 (-1.559; -0.344)	-	1.00	-
TS	-0.146 (-0.729; 0.436)	0.805 (-0.037; 1.647)	2.24 (0.96; 5.19)	0.061
FR	-0.227 (-0.810; 0.356)	0.725 (-0.116; 1.566)	2.06 (0.89; 4.79)	0.091
EBI	-0.266 (-0.850; 0.317)	0.685 (-0.159; 1.529)	1.98 (0.85; 4.61)	0.110
TS+FR	-0.114 (-0.720; 0.491)	0.837 (-0.022; 1.697)	2.31 (0.98; 5.46)	0.056
TS+EBI	-0.761 (-1.420; -0.101)	0.191 (-0.703; 1.084)	1.21 (0.50; 2.96)	0.673
FR+EBI	-0.725 (-1.312; -0.138)	0.227 (-0.622; 1.076)	1.25 (0.54; 2.93)	0.598
TS+FR+EBI	-0.154 (-0.738; 0.429)	0.797 (-0.044; 1.638)	2.22 (0.96; 5.14)	0.063

Based on the factorial design, the absence or presence of different elements of the intervention was explored in relation to the primary and secondary outcomes. Significant positive effects were observed for the proportion screened for those interventions involving TS, FR, TSFR and TSFREBI (table 5). No effects were observed in terms of the proportion of screen positives given advice during the 12-week implementation period (table 6). In terms of the proportion receiving an intervention, significant positive effects were observed for TS, FR, TSFR and TSFREBI, table 7.

This article is protected by copyright. All rights reserved.

Table 5: Mean natural log of proportion screened during 12-week implementation period, mean difference (95% CI), ratio (95% CI) and significance value by presence or absence of factor.

	Mean during 12-week implementation period (95% CI)	Difference versus absent (95% CI)	Ratio versus absent (95% CI)	Sig.
**TS**				
Absent	-3.253 (-4.180; -2.325)	-	1.00	
Present	-2.859 (-3.786; -1.932)	0.394 (0.120; 0.667)	1.48 (1.13; 1.95)	0.05
**FR**				
Absent	-3.398 (-4.306; -2.489)	-	1.00	
Present	-2.705 (-3.613; -1.796)	0.693 (0.444; 0.942)	2.00 (1.56; 2.56)	<0.001
**EBI**				
Absent	-2.951 (-3.852; -2.050)	-	1.00	
Present	-3.155 (-4.056; -2.255)	-0.204 (-0.483; 0.075)	0.82 (0.62; 1.08)	0.150
**TSFR**				
Absent	-3.269 (-4.196; -2.342)	-	1.00	
Present	-2.418 (-3.342; -1.494)	0.851 (0.569; 1.133)	2.34 (1.77; 3.10)	<0.001
**TSEBI**				
Absent	-3.076 (-3.990; -2.162)	-	1.00	
Present	-2.998 (-3.899; -2.077)	0.088 (-0.240; 0.417)	1.09 (0.77; 1.52)	0.596
**FREBI**				
Absent	-3.098 (-4.013; -2.183)	-	1.00	
Present	-2.924 (-3.836; -2.012)	0.174 (-0.149; 0.498)	1.19 (0.86; 1.64)	0.287
**TSFREBI**				
Absent	-3.119 (-4.037; -2.202)	-	1.00	
Present	-2.601 (-3.525; -1.677)	0.518 (0.106; 0.930)	1.68 (1.11; 2.53)	0.014

This article is protected by copyright. All rights reserved.

Table 6: Mean natural log of proportion given brief advice during 12-week implementation period, mean difference (95% CI), ratio (95% CI) and significance value by presence or absence of factor.

	Mean during 12-week implementation period (95% CI)	Difference versus absent (95% CI)	Ratio versus absent (95% CI)	Sig.
**TS**				
Absent	-0.536 (-0.839; -0.232)	-	1.00	
Present	-0.270 (-0.582; 0.043)	0.266 (-0.170; 0.701)	1.30 (0.18; 2.02)	0.229
**FR**				
Absent	-0.509 (-0.823; -0.196)	-	1.00	
Present	-0.309 (-0.614; -0.004)	0.200 (-0.238; 0.638)	1.22 (0.79; 1.89)	0.367
**EBI**				
Absent	-0.352 (-0.661; -0.044)	-	1.00	
Present	-0.461 (-0.772; -0.150)	-0.109 (-0.547; 0.329)	0.90 (0.58; 1.43)	0.624
**TSFR**				
Absent	-0.498 (-0.749; -0.247)	-	1.00	
Present	-0.135 (-0.567; 0.297)	0.363 (-0.137; 0.862)	1.44 (0.87; 2.37)	0.153
**TSEBI**				
Absent	-0.402 (-0.653; -0.152)		1.00	
Present	-0.419 (-0.873; 0.035)	-0.017 (-0.536; 0.503)	0.98 (0.58; 1.65)	0.950
**FREBI**				
Absent	-0.394 (-0.649; -0.139)	-	1.00	
Present	-0.442 (-0.870; -0.013)	-0.048 (-0.547; 0.452)	0.95 (0.58; 1.57)	0.850
**TSFREBI**				
Absent	-0.445 (-0.679; -0.210)	-	1.00	
Present	-0.153 (-0.756; 0.451)	0.292 (-0.356; 0.939)	1.34 (0.70; 2.56)	0.374

This article is protected by copyright. All rights reserved.

Table 7: Mean natural log of proportion given alcohol intervention during 12-week implementation period, mean difference (95% CI), ratio (95% CI) and significance value by presence or absence of factor.

	Mean during 12-week implementation period (95% CI)	Difference versus absent (95% CI)	Ratio versus absent (95% CI)	Sig.
**TS**				
Absent	-4.901 (-5.535; -4.267)	-	1.00	
Present	-4.424 (-5.058; -3.789)	0.477 (0.212; 0.743)	1.61 (1.24; 2.10)	0.001
**FR**				
Absent	-4.984 (-5.615; -4.353)	-	1.00	
Present	-4.331 (-4.961; -3.700)	0.694 (0.401; 0.906)	2.00 (1.49; 2.47)	<0.001
**EBI**				
Absent	-4.597 (-5.225; -3.970)	-	1.00	
Present	-4.721 (-5.349; -4.094)	-0.124 (-0.402; 0.154)	0.88 (0.67; 1.17)	0.380
**TSFR**				
Absent	-4.885 (-5.525; -4.246)	-	1.00	
Present	-3.993 (-4.634; -3.352)	0.893 (0.616; 1.170)	2.44 (1.85; 3.22)	<0.001
**TSEBI**				
Absent	-4.693 (-0.191; 0.454)	-	1.00	
Present	-4.561 (-5.198; -3.925)	0.132 (-0.191; 0.454)	1.14 (0.83; 1.74)	0.420
**FREBI**				
Absent	-4.700 (-5.331; -4.096)	-	1.00	
Present	-4.542 (-5.179; -3.905)	0.158 (-0.164; 0.480)	1.17 (0.85; 1.62)	.334
**TSFREBI**				
Absent	-4.728 (-5.362; -4.095)	-	1.00	
Present	-4.188 (-4.856; -3.520)	0.540 (0.130; 0.950)	1.72 (1.14; 2.58)	0.010

Sensitivity analysis suggested the addition of 0.001 to the observed outcome in order to transform the outcomes had no significant impact on the results.

This article is protected by copyright. All rights reserved.

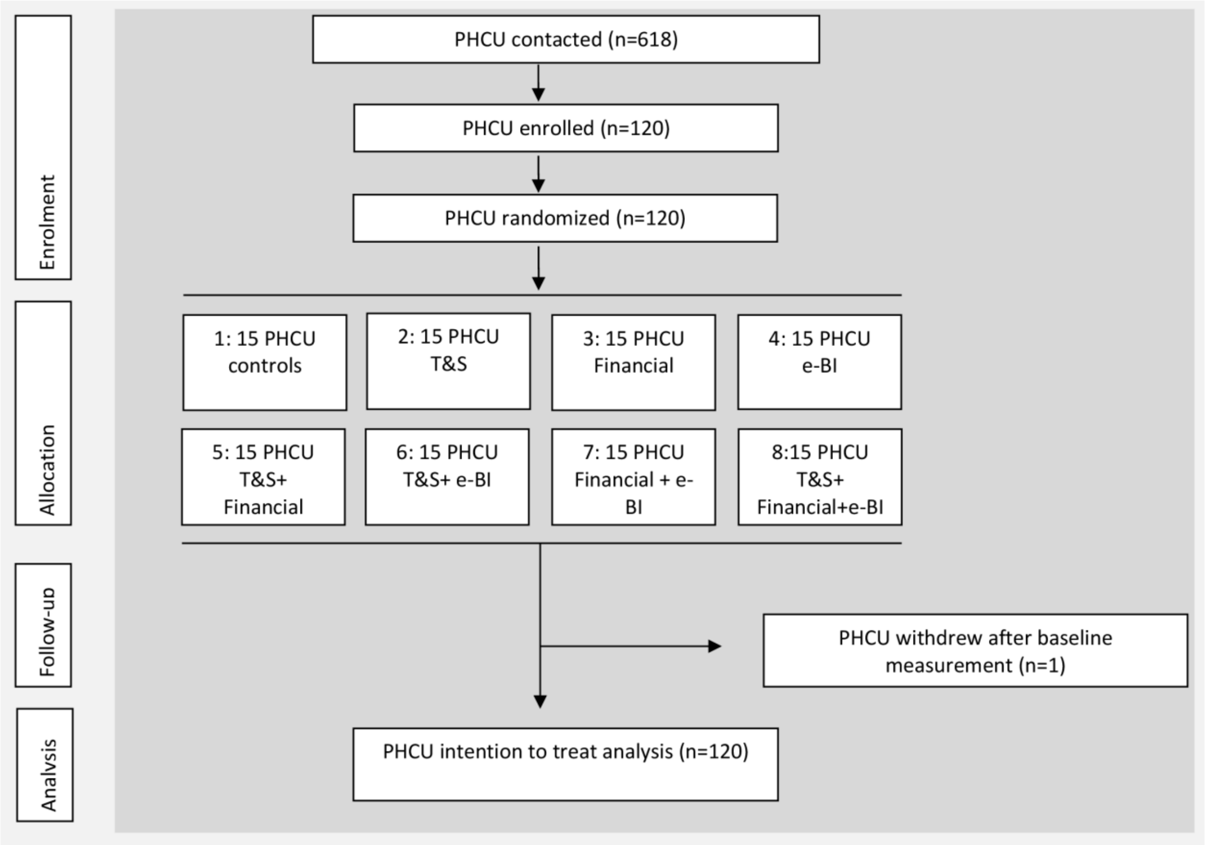

Fig.1. Trial Flow Chart

This article is protected by copyright. All rights reserved.
